# A Case of a Failed Hemiarthroplasty of the Hip Treated by an Extended Trochanteric Osteotomy

**DOI:** 10.7759/cureus.60948

**Published:** 2024-05-23

**Authors:** Abhishek Nair, Archit Gupta, Mukesh O Phalak, Shubhankar Chopra

**Affiliations:** 1 Orthopaedics, Dr. D.Y. Patil Medical College, Hospital and Research Centre, Pimpri, Pune, IND

**Keywords:** bipolar hemiarthroplasty, extended trochanteric osteotomy, failed implant, metallosis, revision joint replacement

## Abstract

Hip bipolar hemiarthroplasty, a widely employed surgical intervention for managing hip fractures and degenerative hip diseases, can pose significant challenges when revisions become necessary due to complications such as implant loosening, instability, or breakage. This case report presents the intricate management of a 58-year-old male who presented with worsening left hip pain a decade after undergoing hip replacement surgery. Despite a thorough preoperative assessment ruling out infection, intraoperative complexities included the necessity for extended trochanteric osteotomy (ETO) to address a broken stem and associated metallosis. Successful revision surgery was meticulously executed, incorporating techniques for implant removal, femoral shaft augmentation, and postoperative rehabilitation. The ensuing discussion explores the multifaceted aspects of failed hemiarthroplasty, emphasizing the critical roles of surgical precision, judicious patient selection, and ongoing research endeavors aimed at refining surgical strategies to optimize patient outcomes. This case underscores the imperative of a multidisciplinary approach and the continued imperative for advancements in surgical methodologies for effectively managing revision hip arthroplasty cases, thus enhancing the quality of patient care in this intricate clinical domain.

## Introduction

Hip bipolar hemiarthroplasty is a commonly performed surgical procedure utilized in the management of hip fractures, degenerative joint diseases such as osteoarthritis, and other conditions affecting the hip joint. This surgical intervention involves replacing the femoral head with a prosthetic component while preserving the patient's acetabulum. While often successful in restoring mobility and alleviating pain, hip hemiarthroplasty is not without its challenges, particularly when complications arise necessitating revision surgery.

Complications associated with hip hemiarthroplasty can include aseptic loosening, implant instability, periprosthetic fractures, and acetabular wear, among others. These complications may manifest in the form of persistent pain, impaired function, or radiographic evidence of implant failure. When conservative measures fail to address these issues, revision surgery becomes imperative to restore proper hip joint function and alleviate patient discomfort.

Revision surgery for failed hip hemiarthroplasty poses unique challenges compared to primary procedures. These challenges stem from factors such as altered anatomy due to prior surgery, compromised bone stock, and the presence of hardware from the initial implantation. Addressing these challenges requires a comprehensive approach that encompasses thorough preoperative evaluation, meticulous surgical technique, and tailored postoperative rehabilitation protocols.

In this article, we present a case report detailing the management of a patient who presented with worsening hip pain years after undergoing hip hemiarthroplasty. We discuss the complexities encountered during revision surgery, including the necessity for extended trochanteric osteotomy (ETO) to address a broken stem and associated metallosis [1]. Additionally, we explore the various factors contributing to implant failure in this case and highlight the importance of surgical precision, patient selection, and ongoing research efforts in optimizing outcomes for revision hip arthroplasty cases. Through this discussion, we aim to provide insights into the challenges and considerations involved in managing failed hip hemiarthroplasty and contribute to the body of knowledge aimed at improving patient care in this complex clinical scenario.

## Case presentation

A 58-year-old male presents with worsening left hip pain for the past 2-3 months, on top of a three-year history. He reports no recent falls or injuries. Notably, he underwent hip replacement surgery 10 years ago and has no other significant medical conditions. Examination revealed a 14-15 cm healed scar along the left hip but no signs of infection or wound breakdown. While attempting to walk, the patient exhibited a limp. Further evaluation of his hip range of motion is detailed in Table 1.

**Table 1:** Range of movements at hip joints

Movement	Left	Right (normal)
Flexion	90	120
Extension	5	20
Abduction	15	40
Adduction	20	25
Internal rotation at 90° flexion	30	45
External rotation at 90° flexion	30	45

**Figure 1:**
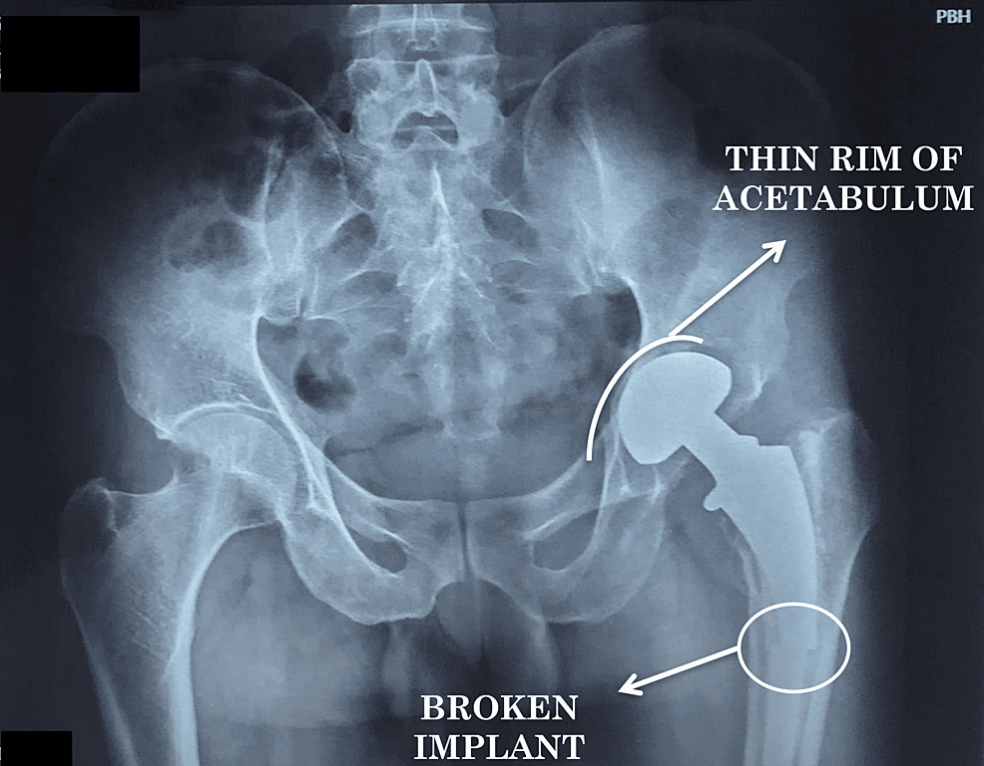
Plan radiograph of the pelvis with both hips showing the thin rim of the acetabulum and the broken implant

**Figure 2:**
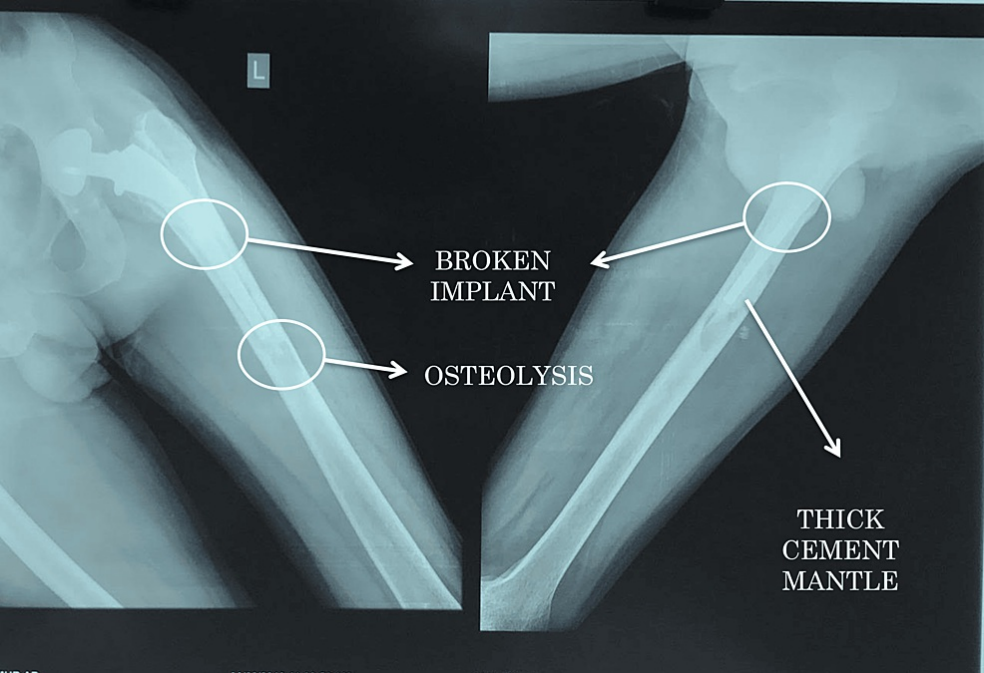
Plain radiograph of the left femur showing the broken implant, osteolysis, and thick cement mantle

**Figure 3:**
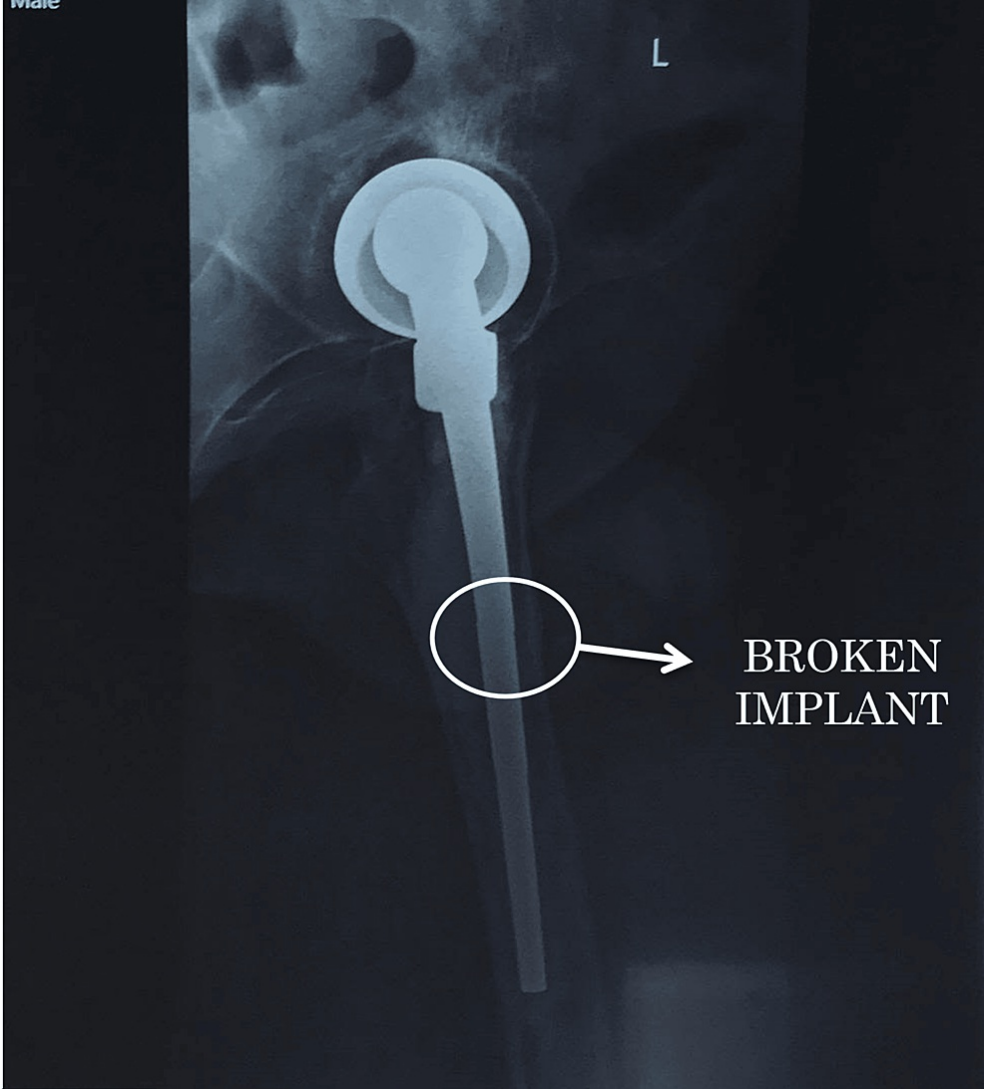
Plain radiograph of the left hip lateral view showing the broken implant

Preoperative radiographs (Figure 1, 2, 3) showed the following features: (1) thin rim of acetabulum and broken implant (Figure 1); (2) broken implant, osteolysis, and thick cement mantle (Figure 2); and (3) lateral view of the broken implant (Figure 3).

In preparation for surgery, routine blood tests were performed to check for infection. The results of these tests are detailed in Table 2.

**Table 2:** Blood investigations All values are found to be within normal range

Investigation	Observed value	Reference range
Hemoglobin	13.8 g/dL	13.2-16.8 g/dL
Total leukocyte count	7400/cu mm	4000-11000/cu mm
C-reactive protein	5.6 mg/L	Less than 10 mg/L
Erythrocyte sedimentation rate	12 mm/h	Less than 20 mm/h

Radiographs were taken to create a template (templating) to determine the appropriate implant size for the surgery. Additionally, a hip aspiration was performed to obtain fluid for culture and rule out any infection, which thankfully showed no bacterial growth. Bone cement was prepared for use during surgery, and consent was obtained for bone grafting in case it becomes necessary during the procedure. It was a challenge to explain the concept of implant breakage and the concept of osteotomy to access the femoral canal and retrieve the metallosis and the broken implant and then reconstructing the femur using cables.

After all necessary preoperative evaluation, the patient was given spinal anesthesia. The patient was then placed in a right lateral position and a standard posterolateral approach was used. An ETO was planned, and a 20 cm incision was made [2]. The sciatic nerve was identified and protected during dissection. Osteotomy was marked longitudinally just lateral to the linea aspera and extending till about 4 cm proximal to the distal extent of the broken stem. The osteotomy fragment was reflected anteriorly along with its soft tissue attachments, and the lateral portion of the stem was visualized. Through manipulation, the head along with the proximal portion of the stem was extracted. The existing cement mantle and the surrounding metallosis were removed piece meal, and the distal fragment of the stem was loosened. Distal fragment was extracted, and any remaining metallosis was removed. After giving a thorough wash, an hydroxyapatite (HA)-coated Wagner stem was placed by standard procedure. Synthes cables were used to augment the femoral shaft.

During surgery (intraoperatively), clinical examination confirmed the absence of infection. The soft tissue surrounding the hip appeared healthy, and the underlying bone structure showed no signs of damage. There were no pockets of pus (sinuses) or drainage, and no collections of fluid indicative of infection. Tissue samples were nonetheless sent for pathology evaluation and culture/sensitivity testing to definitively rule out any underlying infection.

**Figure 4:**
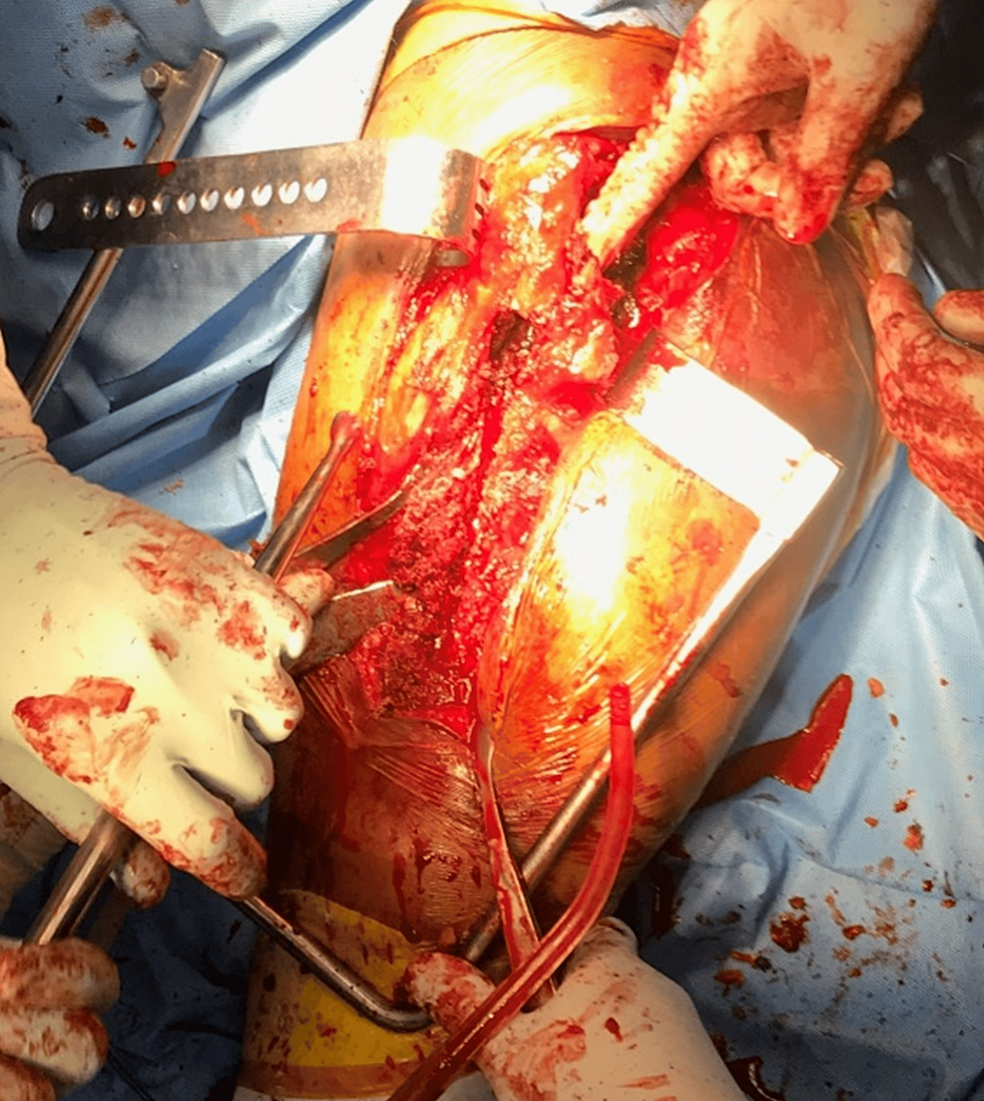
Intraoperative photograph depicting the exposure of the femoral shaft with the greater trochanter being pointed out

**Figure 5:**
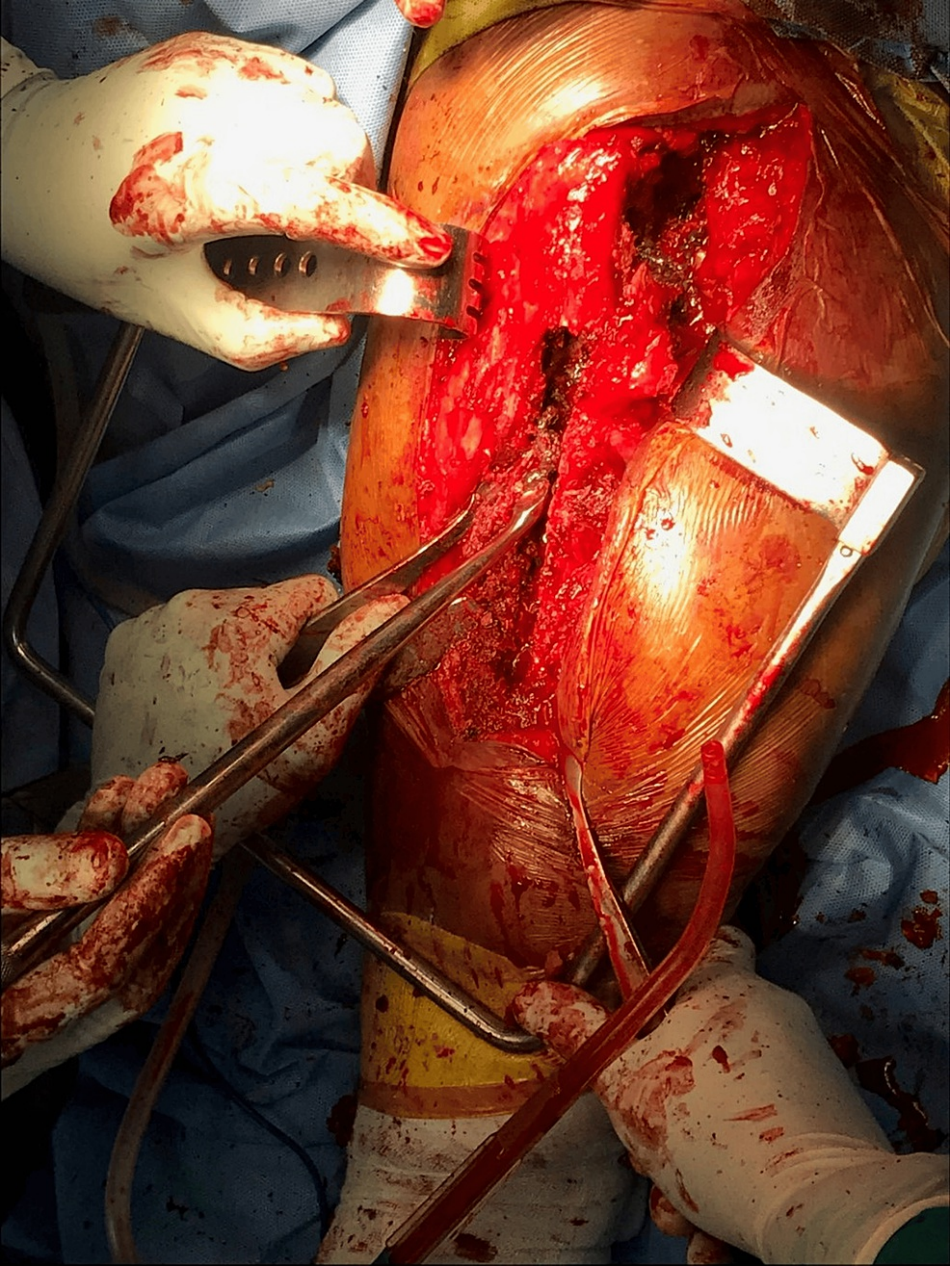
Intraoperative photograph showing the extended trochanteric osteotomy Extended trochanteric esteotomy was done to remove the cement

**Figure 6:**
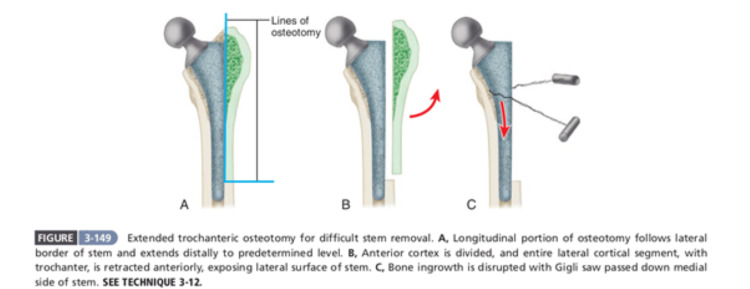
Description of an extended trochanteric osteotomy Source: [2] Published by Elsevier, a signatory to the STM guidelines, and since less than three figures (only one figure) have been used in this research article, notification is not required.

**Figure 7:**
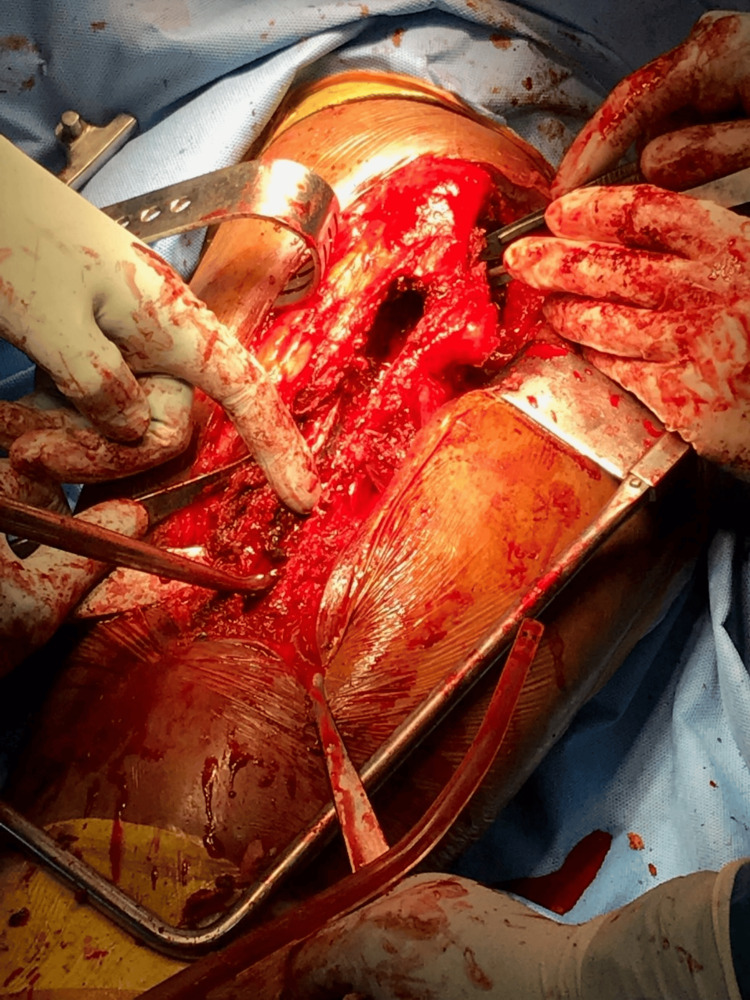
Intraoperative photograph showing the femoral canal after clearing all the cement debris

**Figure 8:**
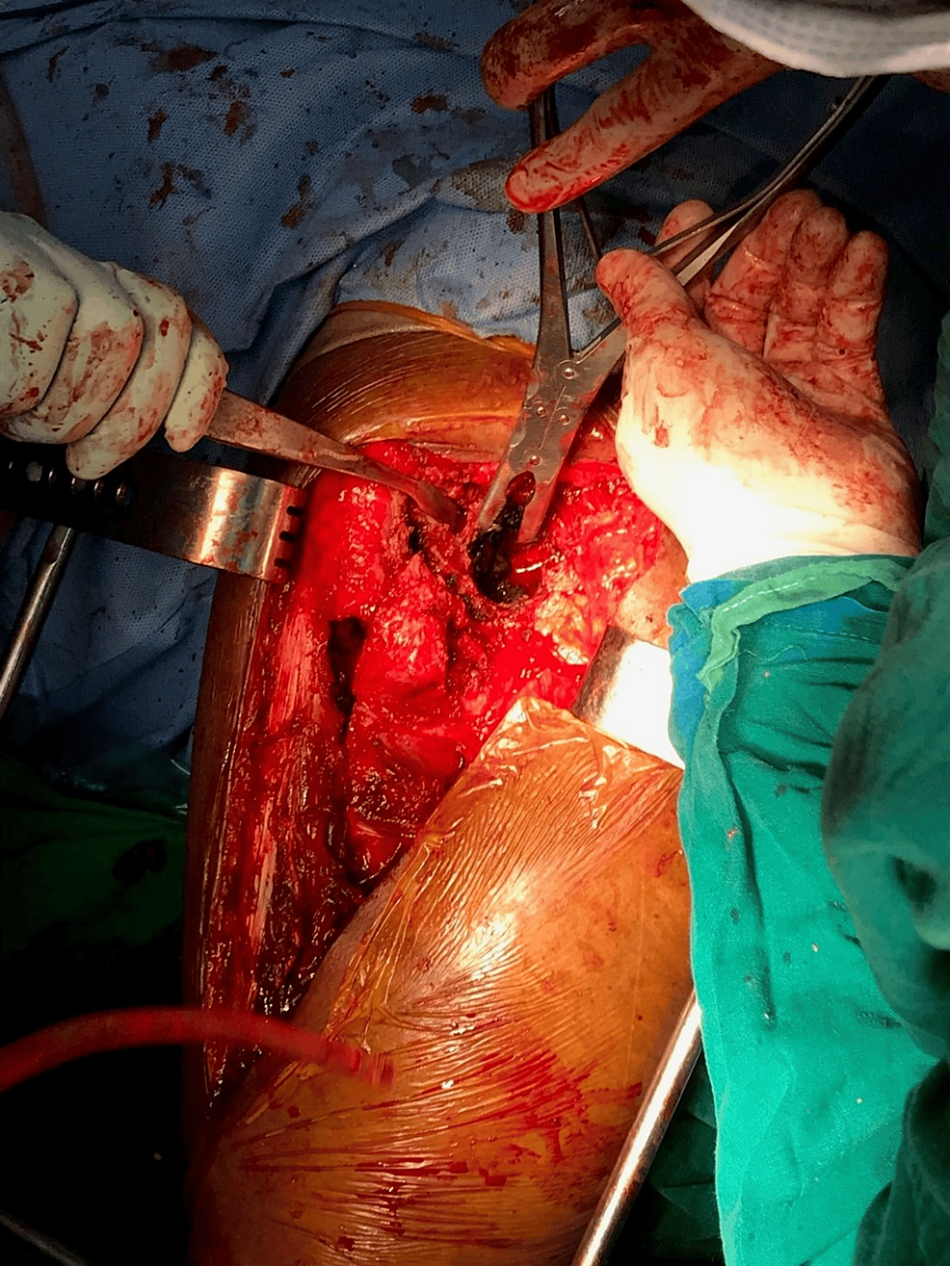
Intraoperative photograph showing the removal of the metallosis

**Figure 9:**
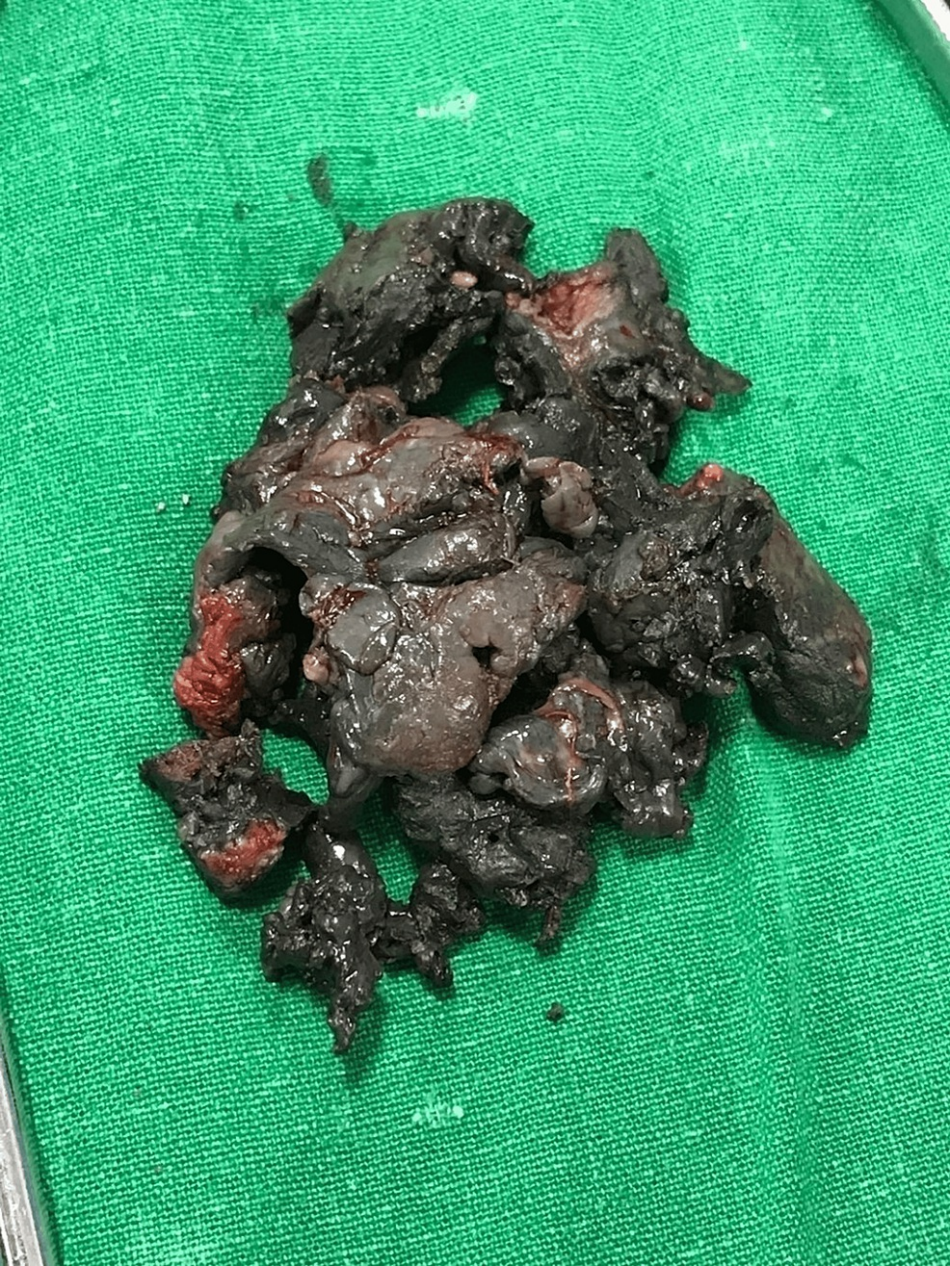
Clinical photograph of the metallosis that was removed

**Figure 10:**
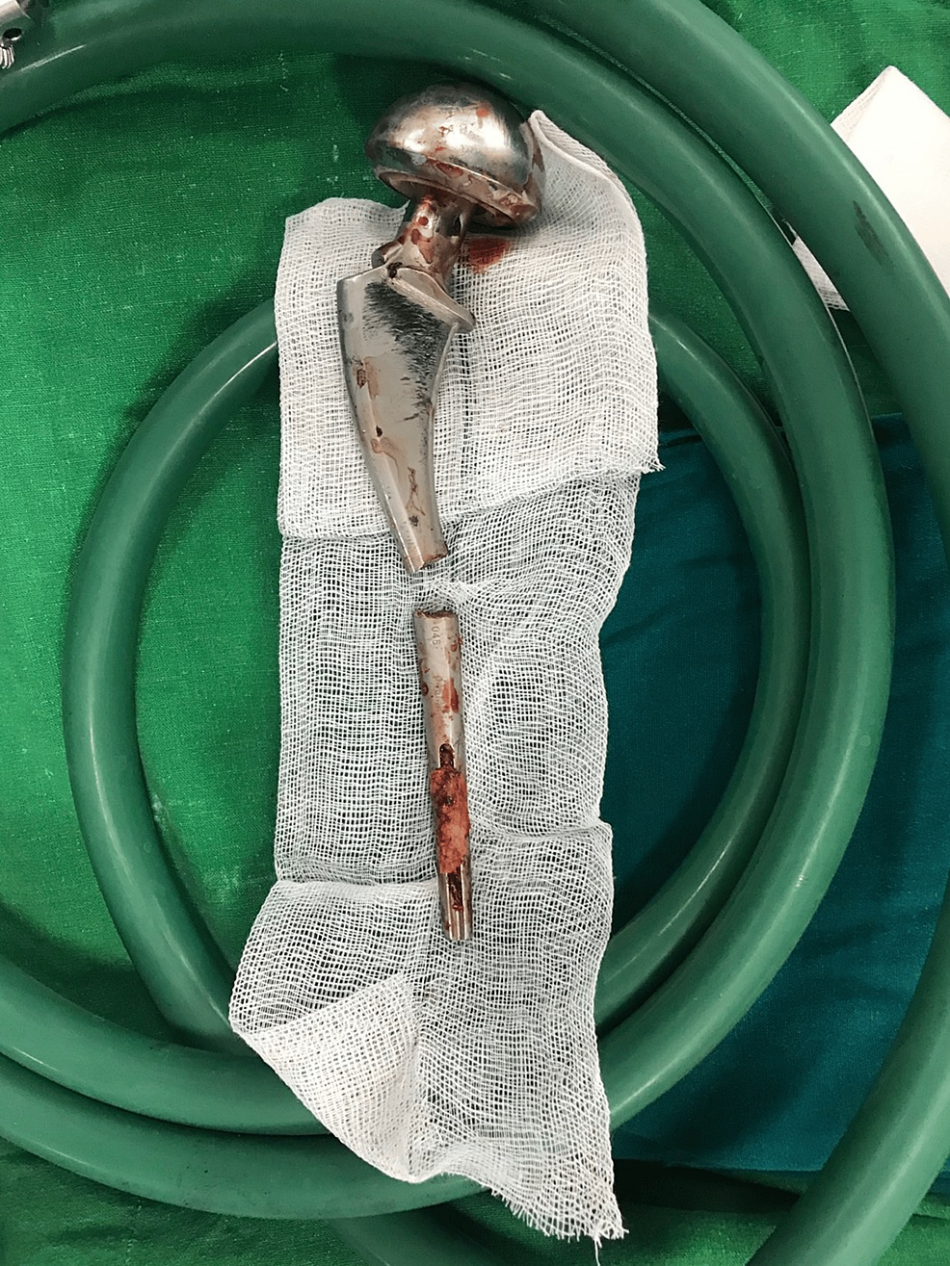
Clinical photograph of the original bipolar implant after removal

**Figure 11:**
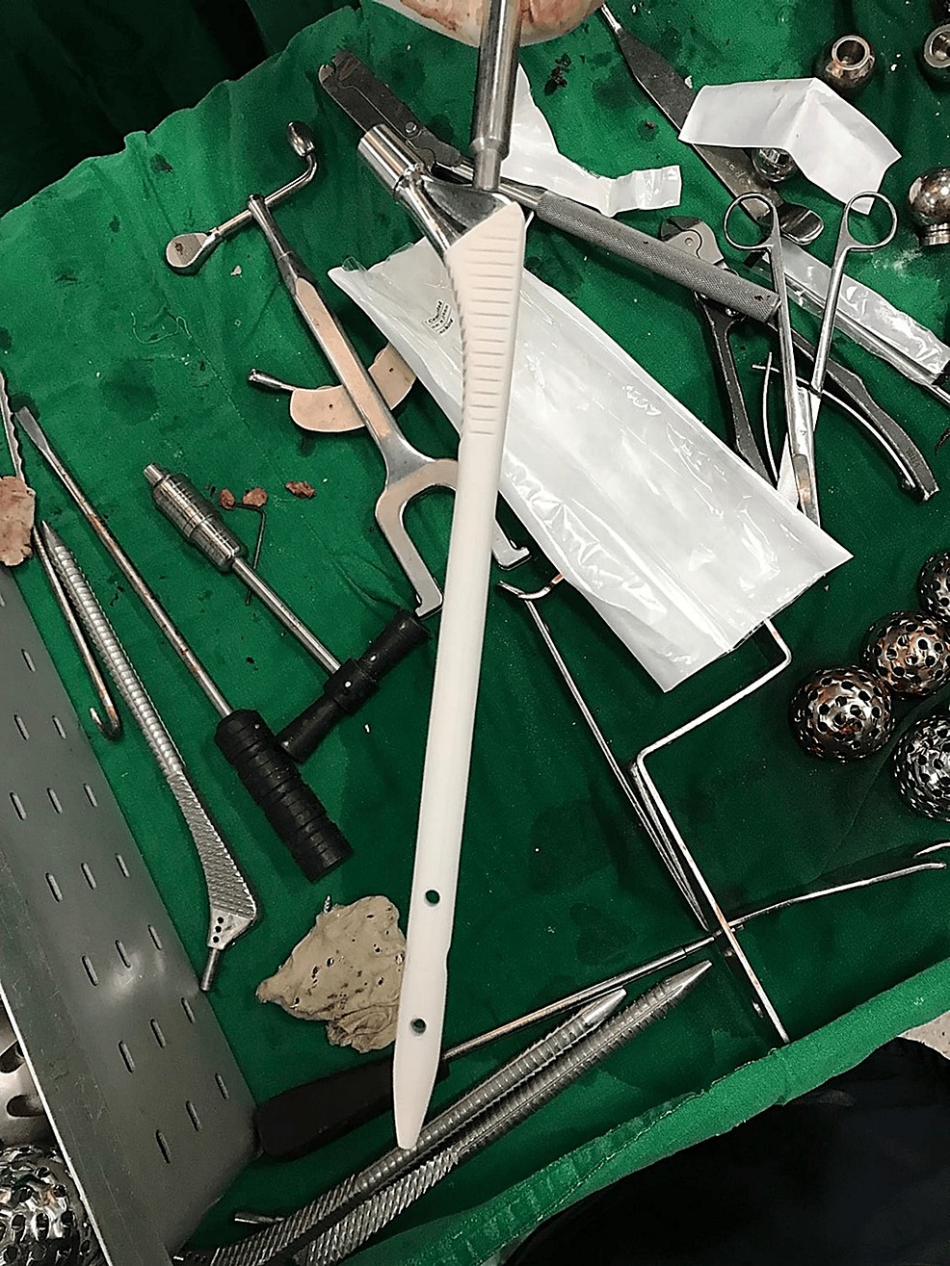
Clinical photograph of the new implant Gen-X long stem, stainless steel

**Figure 12:**
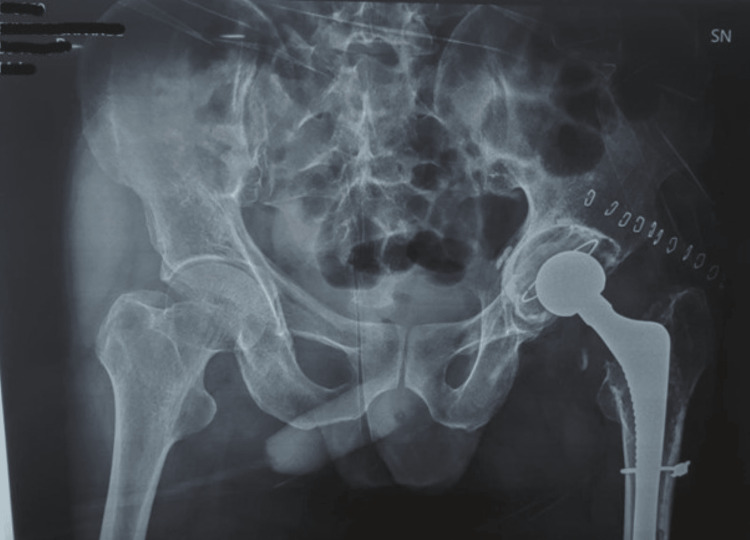
Postoperative plain radiograph of the pelvis with both hips

**Figure 13:**
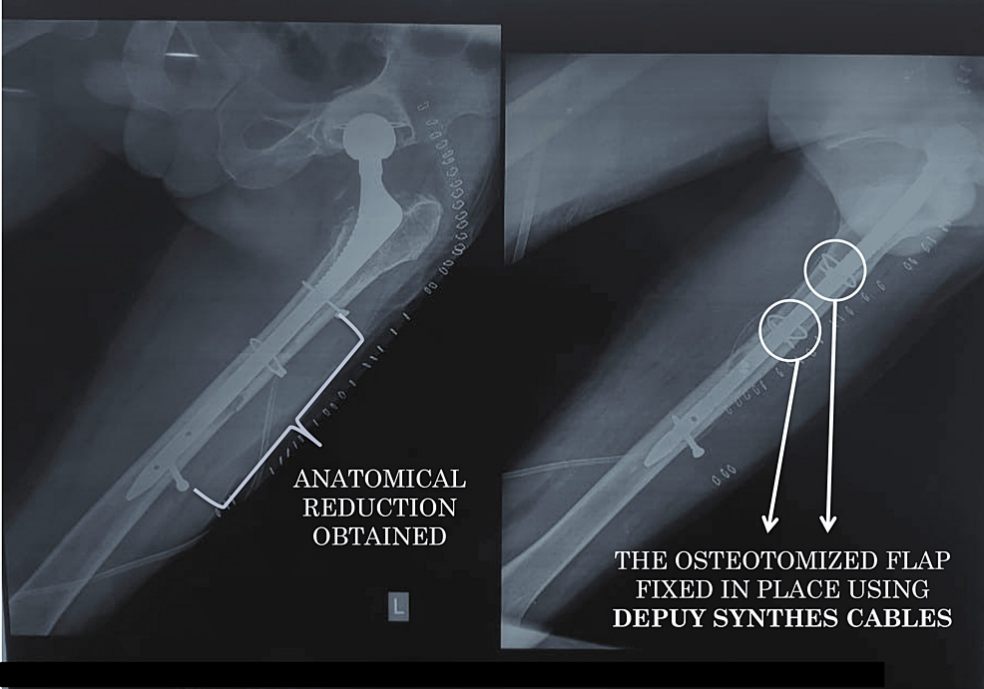
Postoperative plain radiograph of the left femur Anatomical reduction was achieved, and the osteotomy flap was fixed in place with DePuy Synthes Cables

The surgery itself presented several challenges. Ruling out infection definitively was a key concern. Furthermore, meticulous planning of the bone cut (osteotomy) was necessary. Removing the existing cement mantle and implant stem posed a significant hurdle. Reattaching the bone fragments (achieving stable fixation of the osteotomy) while minimizing blood loss and bone loss were crucial aspects. Finally, having a large selection of instruments and implants on hand was essential to address any unforeseen situations. The various steps of surgery have been shown in Figures 4-11. Postoperative radiographs are shown in Figure 12 and Figure 13.

Following surgery, rehabilitation began on the third day, focusing on exercises performed while sitting at the bedside, specifically for the quadriceps and knee range of motion. To promote healing, the patient was instructed to remain non-weight-bearing on the affected hip for the first three weeks, using a walker for ambulation. Additionally, strict avoidance of activities that could stress the joint, such as sitting cross-legged and squatting, was mandated for at least 3-4 weeks postsurgery. Walking was restricted to flat, even surfaces during this time as well. After six weeks, the patient was gradually transitioned to full weight-bearing on the repaired hip.

## Discussion

Our case presentation highlights the complexities associated with failed hip bipolar hemiarthroplasty. While this procedure offers a viable option for specific patient demographics, particularly in fracture management, it is susceptible to various complications necessitating revision surgery. This discussion will delve into the potential causes of failure in our patient's case and explore broader considerations for managing failed bipolar hemiarthroplasty.

One potential explanation for the patient's implant failure lies in aseptic loosening. This phenomenon occurs when the interface between the bone and the prosthesis weakens over time, often due to wear and tear or micromotion [3]. The incidence of implant loosening is as high as 10% after a mean follow-up of 7.1 years [4]. The patient's 10-year history with the implant makes aseptic loosening a strong contender, especially considering the absence of any recent trauma or infection.

Another possibility is implant instability, which can arise from improper component sizing, inadequate bone stock, or periprosthetic fractures [5]. While the preoperative workup likely addressed sizing, the quality of the underlying bone, potentially compromised after 10 years with an implant, could have contributed to instability.

The incidence of implant breakage is around 9% in previously published data [5]. Fatigue fracture of the stem in hemiarthroplasty is sparsely reported in the literature [6,7]. Distal bonding of the stem, varus alignment of the stem, obesity, and lack of proximal support at the calcar femorale causing cantilever effect and bending mode of failure are cited as the causes for fatigue fracture of the stem. Broken stems are extracted by endo-femoral or transfemoral approach [8]. In the endo-femoral approach, the stems are extracted using an extraction hook, long disc punch, or hollow mill under fluoroscopy guidance, but it is time-consuming.

ETO was necessitated in our case due to the broken stem and long interval from index surgery. ETO provides several advantages such as providing superior visualization of the acetabulum and proximal femur, facilitating meticulous removal of existing implants, and thorough assessment of the bone quality. ETO allows for easier extraction of well-fixed implants, reducing the risk of intraoperative fractures and minimizing soft tissue damage. In cases of severe bone loss or malpositioned components, ETO enables precise restoration of the hip center, optimizing biomechanics and implant stability. By providing greater access to the femoral canal and acetabulum, ETO facilitates accurate placement and fixation of revision components, thereby improving long-term outcomes. Careful handling of the trochanteric fragment during ETO preserves the integrity of surrounding soft tissues, promoting faster rehabilitation and reducing the risk of postoperative complications.

ETO plays a pivotal role in the armamentarium of the revision hip arthroplasty surgeon, offering enhanced exposure, improved component fixation, and better outcomes in challenging cases. While associated with potential complications, judicious patient selection, meticulous surgical technique, and appropriate adjunctive measures can mitigate these risks, making ETO an indispensable tool in the pursuit of successful revision hip arthroplasty.

Furthermore, the possibility of acetabular wear, although less common in bipolar hemiarthroplasty compared to total hip arthroplasty, cannot be entirely ruled out [9]. Long-term wear of the polyethylene liner within the acetabular cup could have played a role in the patient's pain and functional limitations.

The successful revision surgery emphasizes the importance of meticulous intraoperative assessment and management strategies. Ruling out infection definitively, as demonstrated in this case, is crucial to guide implant selection and surgical approach. Additionally, meticulous planning of the osteotomy and careful removal of the existing implant with minimal bone loss are vital for a successful outcome.

Our patient's case also underscores the significance of thorough preoperative evaluation. Patient selection for bipolar hemiarthroplasty is critical, considering factors like bone quality, activity level, and potential for future revisions. Additionally, surgical technique plays a key role in long-term implant survival, highlighting the importance of experienced surgeons familiar with the nuances of both hemiarthroplasty and revision surgery. A limitation that we found was that other rapid tests like leucocyte esterase test and alpha defensin test could have been employed for further confirmation.

## Conclusions

In conclusion, failed hip bipolar hemiarthroplasty presents a complex clinical scenario. Understanding the potential causes of failure, meticulous surgical technique, and careful patient selection are paramount for achieving optimal outcomes in revision surgery. Future research focusing on improved implant designs, wear-resistant materials, and surgical techniques tailored for revision procedures can further enhance patient outcomes in this challenging area.
